# Inhalable nanocatchers for SARS-CoV-2 inhibition

**DOI:** 10.1073/pnas.2102957118

**Published:** 2021-07-02

**Authors:** Han Zhang, Wenjun Zhu, Qiutong Jin, Feng Pan, Jiafei Zhu, Yanbin Liu, Linfu Chen, Jingjing Shen, Yang Yang, Qian Chen, Zhuang Liu

**Affiliations:** ^a^Institute of Functional Nano and Soft Materials, Jiangsu Key Laboratory for Carbon-Based Functional Materials and Devices, Soochow University, Suzhou 215123, China;; ^b^Department of Thoracic Surgery, Shanghai Pulmonary Hospital, Tongji University School of Medicine, Shanghai 200433, China;; ^c^School of Materials Science and Engineering, Tongji University, Shanghai 201804, China;; ^d^Macao Institute of Materials Science and Engineering, Macau University of Science and Technology, Taipa 999078, China

**Keywords:** SARS-CoV-2, virus inhibitor, hACE2-containing nanocatchers, inhalation, mucoadhesive

## Abstract

The global coronavirus disease 2019 (COVID-19) pandemic, caused by severe acute respiratory syndrome (SARS)–like coronavirus (SARS-CoV-2), presents an urgent health crisis. More recently, an increasing number of mutated strains of SARS-CoV-2 have been identified globally. Such mutations, especially those on the spike glycoprotein to render its higher binding affinity to human angiotensin-converting enzyme II (hACE2) receptors, not only resulted in higher transmission of SARS-CoV-2 but also raised serious concerns regarding the efficacies of vaccines against mutated viruses. Since ACE2 is the virus-binding protein on human cells regardless of viral mutations, we design hACE2-containing nanocatchers (NCs) as the competitor with host cells for virus binding to protect cells from SARS-CoV-2 infection. The hACE2-containing NCs, derived from the cellular membrane of genetically engineered cells stably expressing hACE2, exhibited excellent neutralization ability against pseudoviruses of both wild-type SARS-CoV-2 and the D614G variant. To prevent SARS-CoV-2 infections in the lung, the most vulnerable organ for COVID-19, we develop an inhalable formulation by mixing hACE2-containing NCs with mucoadhesive excipient hyaluronic acid, the latter of which could significantly prolong the retention of NCs in the lung after inhalation. Excitingly, inhalation of our formulation could lead to potent pseudovirus inhibition ability in hACE2-expressing mouse model, without imposing any appreciable side effects. Importantly, our inhalable hACE2-containing NCs in the lyophilized formulation would allow long-term storage, facilitating their future clinical use. Thus, this work may provide an alternative tactic to inhibit SARS-CoV-2 infections even with different mutations, exhibiting great potential for treatment of the ongoing COVID-19 epidemic.

## Significance

The recently emerged SARS-CoV-2 variants are more transmissible, which brings new challenges to vaccine treatment. There is an urgent global need for alternative strategies that could effectively and rapidly prevent the infection of various SARS-CoV-2 variants. Herein, we design human angiotensin-converting enzyme II (hACE2)–containing nanocatchers (NCs) derived from genetically engineered cells stably expressing hACE2 as the competitor with host cells for virus binding to protect cells from SARS-CoV-2 infection. An inhalable formulation fabricated by NCs and the mucoadhesive excipient hyaluronic acid could significantly prolong the retention of NCs in the lung and exhibits potent pseudovirus inhibition ability in an hACE2-expressing mouse model. Importantly, the inhalable NCs in the lyophilized formulation allow long-term storage, facilitating their future clinical use.

The expanding coronavirus disease 2019 (COVID-19) pandemic, caused by severe acute respiratory syndrome coronavirus 2 (SARS-CoV-2), has infected more than 200 million people and killed over 3 million, and the numbers are still rapidly rising in April 2021 (1, 2). Like other zoonotic coronaviruses, SARS-CoV-2 with the surface spike (S) glycoprotein binds with the receptor human angiotensin-converting enzyme II (hACE2) for cell entry and infection (3–6). Moreover, the S protein also undergoes mutations all the time to optimize its binding affinity and binding mode with ACE2 receptors, which may alter pathogenesis, virulence, and transmissibility (7–9). The spike aspartic acid–614 to glycine (D614G), the dominant SARS-CoV-2 mutational form globally, showed obviously increased binding efficiency with ACE2 receptor during the virus infection process (10, 11). Moreover, a new variant strain of SARS-CoV-2, B.1.1.7 (also known as VOC 202012/01), with a high number of genetic mutations has been found in London and is spreading worldwide (12, 13). The binding affinity of S protein of B.1.1.7 to hACE2 receptor is increased by 1,000 times, and it exhibits 70% more transmissible ability than the previously discovered SARS-CoV-2 (14).

Vaccines, one of the effective strategies to prevent the spread of infectious diseases by reducing morbidity and mortality, have attracted wide attention since the outbreak of COVID-19. At present, there are more than 314 SARS-CoV-2 vaccines in the research and development stage in the world, 89 of which have entered the clinical trial stage, and multiple research and development technologies are being promoted in parallel to promote vaccine development (15). Excitingly, thirteen vaccines have been approved for clinical use in different countries, including mRNA vaccines, virus-vectored vaccines, inactivated virus vaccines, and protein subunit vaccines (https://vac-lshtm.shinyapps.io/ncov_vaccine_landscape/#https://vac-lshtm.shinyapps.io/ncov_vaccine_landscape/). The current vaccines primarily protect the host against infection by producing neutralizing antibodies specific for the surface S protein (16–18). However, mutation of the S protein may possibly limit the efficiency of these vaccines (19–21). For example, the neutralizing activity of the serum of volunteers who have received either the Moderna (mRNA-1273) or Pfizer-BioNTech (BNT162b2) vaccines against the South Africa mutant strain (B.1.351) have been proven to be reduced (22, 23). Thus, novel strategies that could effectively and rapidly prevent the infection of SARS-CoV-2 with different mutations take on a renewed urgency in this period for COVID-19.

Considering that the infectivity of SARS-CoV-2 depends on the binding with the entry receptor hACE2, recent studies have investigated the potential of cellular nanovesicles (NVs) containing hACE2 to compete with host cells for SARS-CoV-2 binding to protect host cells from the infection of SARS-CoV-2 (24, 25). How to improve the therapeutic efficiency of such neutralizing cellular NVs is still an important issue for COVID-19 treatment. Herein, the hACE2 nanocatchers (NCs) were fabricated from genetically engineering human embryonic kidney 293T cells with hACE2 as the neutralizing NCs to protect the host from the infection of SARS-CoV-2. As expected, such hACE2-containing NCs displayed excellent binding affinity to the coronavirus and its mutant and inhibited their usual infection ability in vitro. Moreover, in view of the fact that the effective retention of cellular NVs in the lungs after inhalation may be a prerequisite for inhibiting SARS-CoV-2 infection of the lung, we try to seek effective mucoadhesives to increase epithelial contact, decrease mucociliary transport rate, and finally, maintain the retention of NCs in the lungs for a longer duration after pulmonary drug delivery. Excitingly, it was found that the mucoadhesive excipient hyaluronic acid (HA) with high biocompatibility introduced here was able to significantly improve the retention of NCs in the lung, exhibiting potent SARS-CoV-2 pseudovirus inhibition ability in the mouse model with replication defective adenovirus encoding for hACE2. More importantly, NCs in the lyophilized formulation were fabricated with the assistance of cryoprotectant sucrose, increasing the feasibility of clinical use including transport and long-term storage.

## Results

**Fig. 1.**
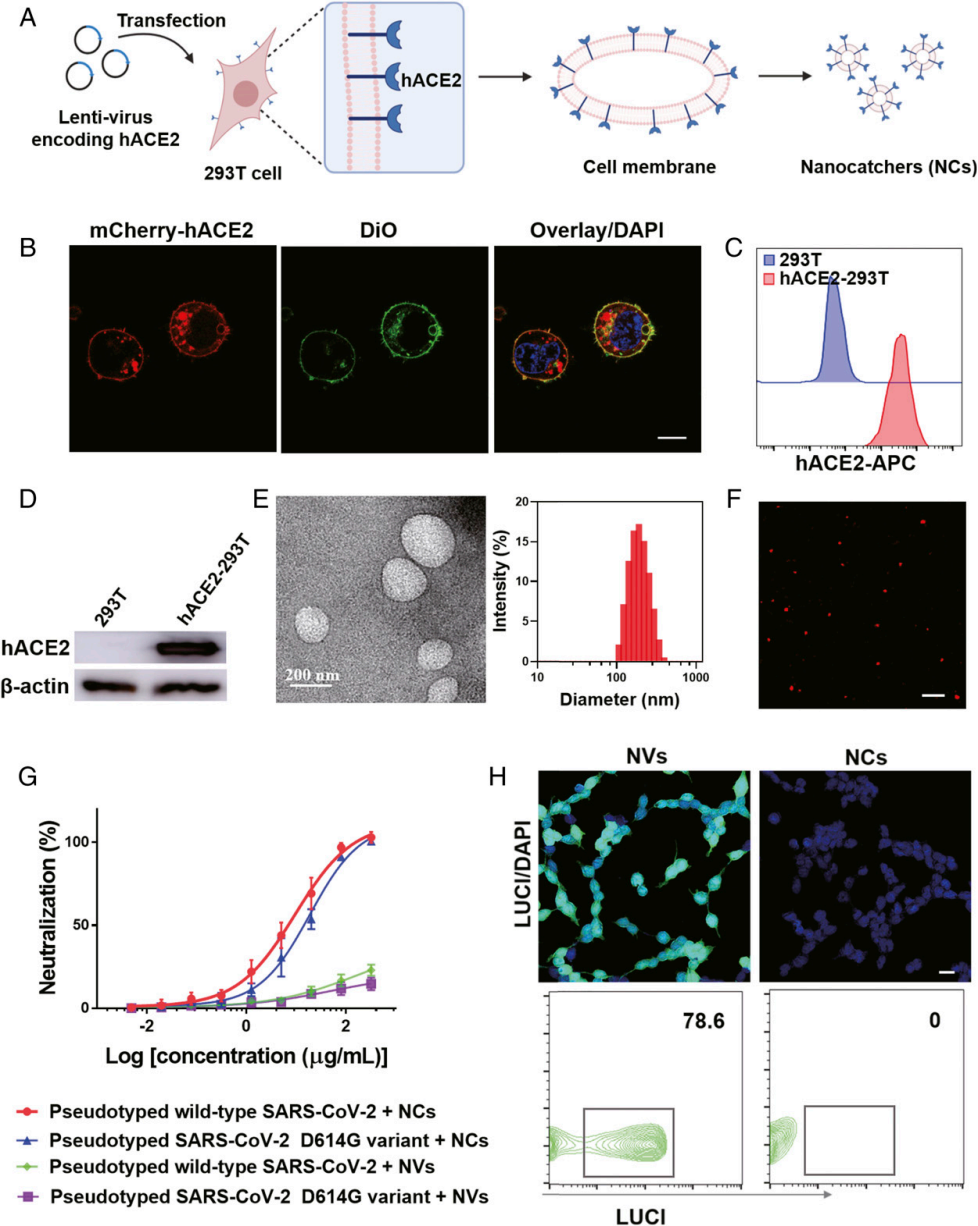
Fabrication and characterization of hACE2-containing NCs. (*A*) Schematic showing the preparation of NCs. (*B*) Confocal image of 293T cells stably expressing hACE2. DiO dye was used to indicate the cell membranes. (Scale bar, 10 µm.) (*C*) Flow cytometry and (*D*) Western blotting analysis of the expression of hACE2 in 293T cells. (*E*) TEM image (*Left*) and average hydrodynamic size of NCs derived from hACE2-293T cells (*Right*). (*F*) Confocal image of NCs. (Scale bar, 1 µm.) (*G*) Pseudotyped wild-type SARS-CoV-2 and SARS-CoV-2 D614G variant neutralization curves by NCs or NVs, which were collected from 293T cells with hACE2 over-expression or wide-type 293T cells, respectively. The data are presented as mean ± SEM (*n* = 3). (*H*) Representative confocal images (*Top*) and flow cytometry data (*Bottom*) of hACE2-293T cells infected by pseudovirus with NVs or NCs. (Scale bar, 20 µm.)

The fabrication process of hACE2-containing NCs is depicted in Fig. 1*A*. Specifically, 293T cells were infected with lentivirus encoding hACE2 and selected by puromycin to genetically engineer hACE2 stable expressing 293T (hACE2-293T) cells. An mCherry-tagged hACE2-encoding lentivirus was employed to indicate the expression of hACE2. As indicated in Fig. 1*B*, the signal from mCherry-hACE2 was clearly observed, and it was colocalized with the cell membrane dye 3,3′-dioctadecyloxacarbocyanine perchlorate (DiO), proving that hACE2 was successfully expressed in the cell membranes. The high expression of hACE2 was confirmed by flow cytometry and Western blotting analysis (Fig. 1 *C* and *D*), and the high expression level was sustained at different passage numbers of hACE2-293T cells (*SI Appendix*, Fig. S1). Subsequently, the NCs were derived following the reported literature protocol with slight modifications (26). As observed in the transmission electron microscopy (TEM) imaging and dynamic light scattering (DLS) measurement (Fig. 1*E*), the obtained NCs showed a spherical morphology with an average diameter around 200 nm and a zeta potential of approximate −15.5 mV. Moreover, red signals from the mCherry-hACE2 also were observed in the obtained NCs by confocal imaging, indicating that the high-density hACE2 was remained in the obtained NCs (Fig. 1*F*).

Encouraged by the successful preparation of hACE2-containing NCs, we then evaluated their neutralization activity of SARS-CoV-2 by binding with the S glycoprotein using vesicular stomatitis virus (VSV)–based pseudotyped SARS-CoV-2. The pseudovirus containing the S protein of coronaviruses and the luciferase (LUCI) reporter gene is a reliable and safe tool to screen and characterize new drugs with anticoronavirus infection activities. Before investigating the virus neutralization ability, a standard methyl thiazolyl tetrazolium (MTT) assay was carried out to evaluate the cytotoxicity of NCs on human umbilical vein endothelial (HUVEC) and 293T cells. No obvious cytotoxicity was observed for both cells even at the highest tested dose (*SI Appendix*, Fig. S2), indicating the inherent biocompatibility of NCs. Then, a neutralization titration assay was performed by coincubation of pseudovirus and diluted NCs and then transferring the mixture to hACE2-293T monolayers for infection. Considering that the SARS-CoV-2 D614G variant exhibits a stronger infectious ability than the wild type and has become globally prevalent, the neutralization ability of NCs against the pseudotyped SARS-CoV-2 D614G variant was investigated. As shown in Fig. 1*G*, the NCs bound to the D614G variant exhibited a half maximal inhibitory concentration (IC_50_) at 16.3 μg/mL, comparable with that against the wild-type pseudovirus (9.5 μg/mL), demonstrating that our hACE2-containing NCs could act as a potent competitor with host cells for virus binding to protect cells from SARS-CoV-2 infection regardless of viral mutations. Notably, 293T cell–derived NVs without the overexpression of hACE2 showed rather weak neutralization ability against either wild-type SARS-CoV-2 or the D614G variant (Fig. 1*G*). In the confocal imaging, no signal from the anti-LUCI antibody was observed in cells incubated with NCs and pseudovirus, while strong green fluorescence signals were detected when using no hACE2-expressing 293T cell–derived NVs and pseudovirus, indicating that hACE2 NCs could act as efficient neutralization agents by competitive binding to the S protein and inhibit virus infection (Fig. 1*H*). Then, the flow cytometry results further confirmed that the high density of hACE2 in the NC membrane could protect SARS-CoV-2–sensitive cells from infection (Fig. 1*H*). Thus, the NCs developed in this work are able to compete with host cells for SARS-CoV-2 binding to inhibit the viral infection.

Noninvasive inhalation of drug-loaded nanoparticles is a promising strategy for the treatment of pulmonary diseases because of its low systemic adverse effects, ease of administration, and excellent patient compliance (26, 27). Nonetheless, the airway mucus plays a vital role in the clearance of foreign bodies, including nanodrugs, which usually leads to undesirable therapeutic results (28, 29). Mucoadhesives are popular candidates to prolong the residence time and bioavailability of drugs in the lung by decreasing the mucociliary transport rate (MTR) (30, 31). In view of NCs with high surface expression of hACE2 and negative charge, three mucoadhesive excipients—including poly(vinyl alcohol) (PVA), poly(vinyl pyrrolidone) (PVP), and HA—were employed here to evaluate their feasibility to enhance the retention of NCs in the lung. Then, NCs were labeled with the fluorescent dye Cy5.5 to investigate their distribution in the lung.

**Fig. 2.**
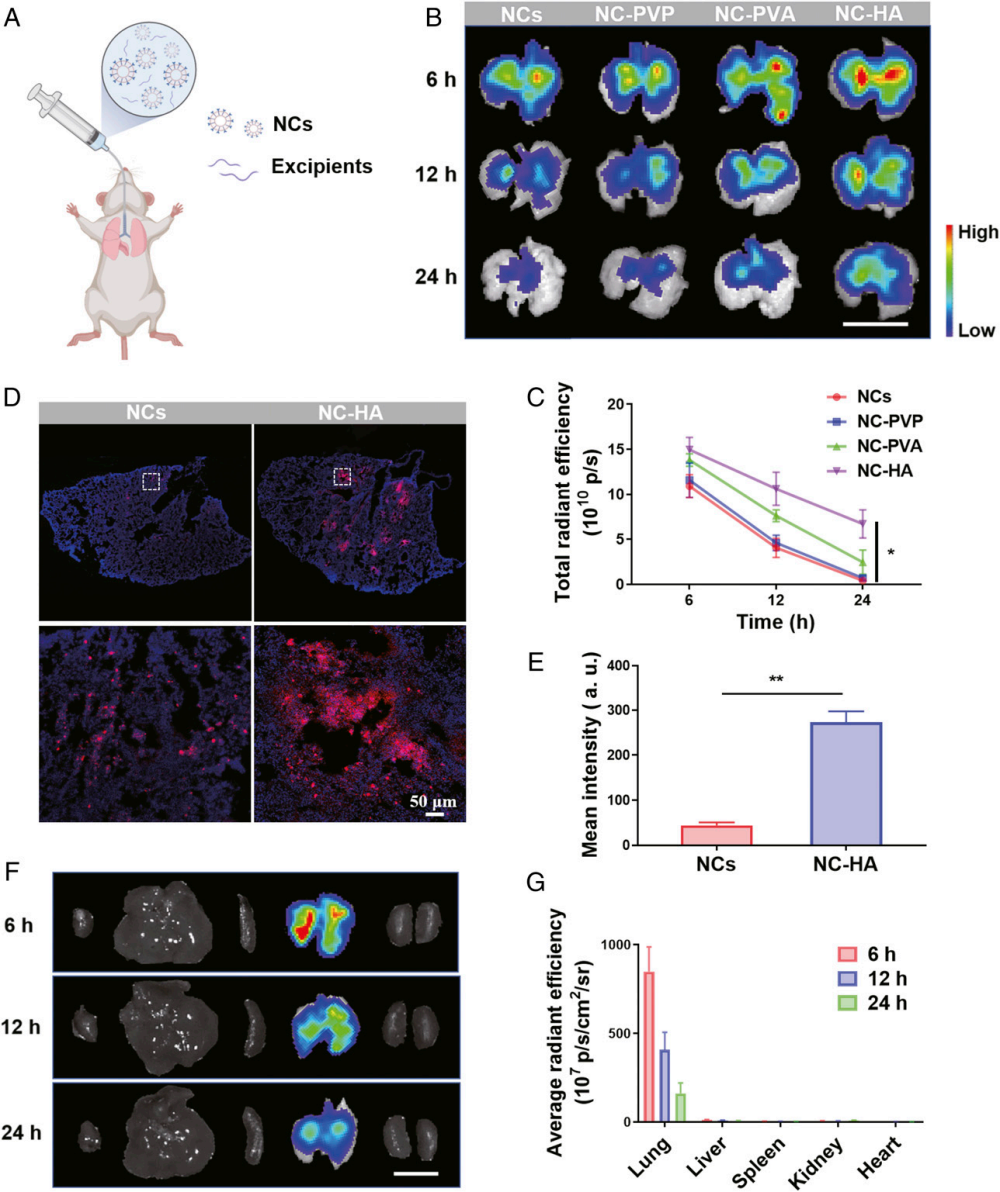
Improved retention of hACE2-containing NCs in vivo. (*A*) Scheme of inhalation of NCs containing excipients. (*B*) Fluorescence IVIS images and (*C*) quantified fluorescence analysis of lung collected at 6, 12, and 24 h postinhalation from mice treated with Cy5.5-labeled NCs in different formulations. (Scale bar, 1 cm.) (*D*) Representative confocal images and (*E*) quantification analysis of Cy5.5-labeled NCs and Cy5.5-labeled NC/HA in the left lobar of lung at 24 h postinhalation. The pictures below are the corresponding enlarged image in the white dotted frame. (Scale bar, 50 μm.) Experiments were repeated three times. (*F*) Ex vivo images showing the biodistribution of Cy5.5-labeled NC/HA. (Scale bar, 1 cm.) (*G*) Corresponding quantitative data of *F*. All data are presented as mean ± SEM (*n* = 5). The data are analyzed by Tukey’s multiple comparisons test. *P < 0.05; **P < 0.01.

Following inhalation of the mixtures of Cy5.5-labeled NCs and various types of excipients (Fig. 2*A*), the lung tissues of mice that inhaled different formulations were explanted and monitored at 6, 12, and 24 h postinhalation using an in vivo imaging system (IVIS) (Fig. 2*B*). Excitingly, with the assistance of HA and PVA, NCs exhibited greatly improved retention ability. It was discovered that the mixture of NCs and HA showed the best retention effect, as the fluorescence signals of Cy5.5 from NCs in the mice treated with NC–HA remained the strongest at all time points (Fig. 2*C*). The excellent retention effect of NC–HA was further substantiated by the confocal imaging of lung sections, in which a remarkable fluorescence signal associated with NCs was found mainly in the primary bronchus, whereas weak fluorescence distributed in the terminal bronchiole was observed in the free NC–treated group (Fig. 2 *D* and *E*), demonstrating that the mucoadhesive excipient HA played an indispensable role in protecting NCs from rapid clearance. Subsequently, the biodistribution of such an NC–HA complex in different major organs at different time points after inhalation was assessed. As shown in the fluorescence IVIS images, compared with other organs, the lung exhibited the strongest fluorescence signal at different time points, and it was distributed throughout the lung tissue, further indicating the prolonged residence time and improved bioavailability of NCs in the lung with the help of mucoadhesive excipient HA (Fig. 2 *F* and *G*). Meanwhile, the addition of HA induced negligible impacts on the size, zeta potential, and IC_50_ value associated with pseudovirus neutralization of NCs, demonstrating that NCs maintained their surface property and neutralization ability in the mixture (*SI Appendix*, Fig. S3). Thus, such an NC–HA complex may be the ideal agnostic to SARS-CoV-2 mutations to protect the host cells from infection after inhalation.

**Fig. 3.**
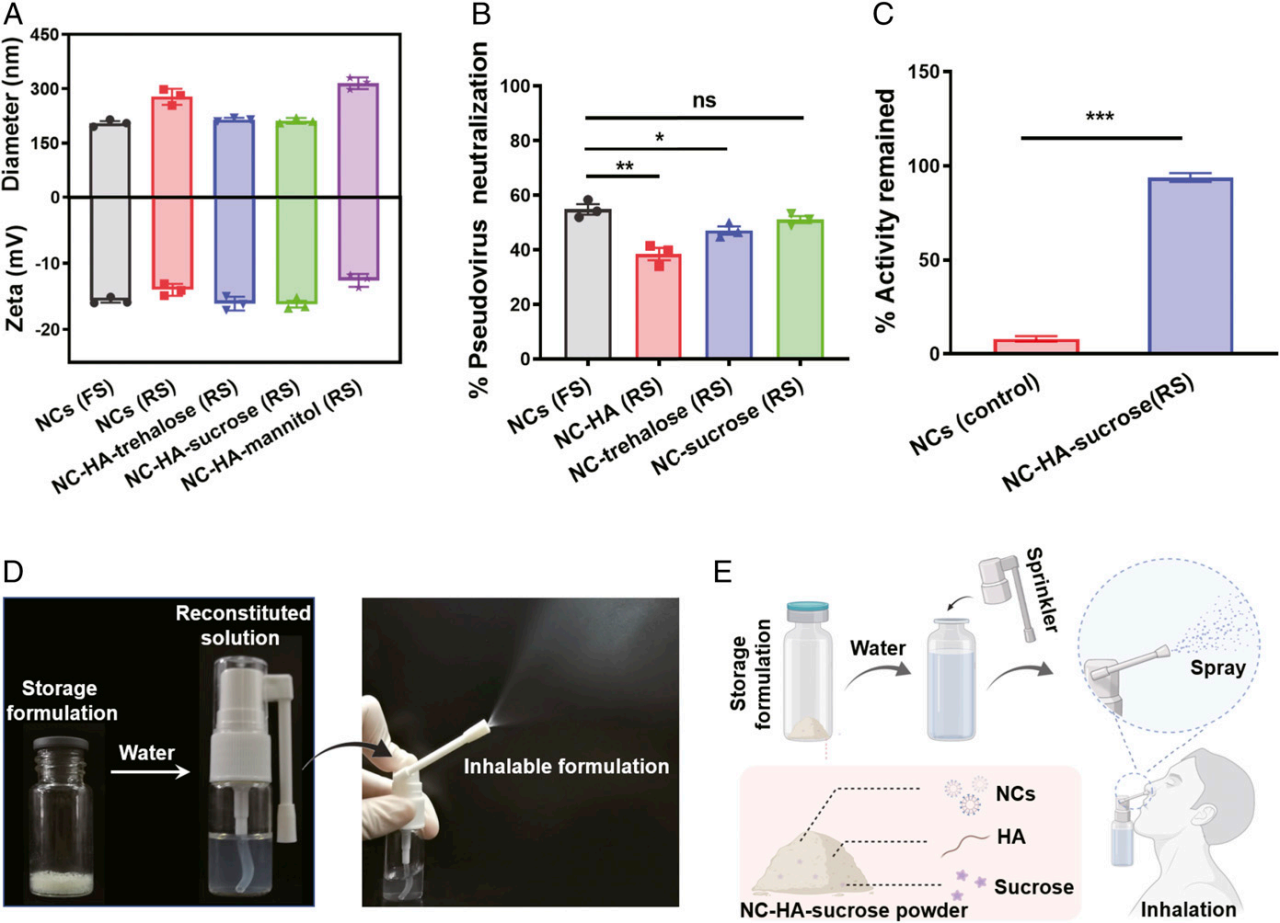
Storage formulation of hACE2-containing NCs. (*A*) Size and zeta potential of freshly prepared solution (FS) of NCs and reconstituted solution (RS) of lyophilized NCs and NC–HA with different cryoprotectants. (*B*) SARS-CoV-2 pseudovirus neutralization efficiency of freshly prepared NCs, reconstituted NC-HA, and NC–cryoprotectant (equivalent NCs concentration, 10 μg/mL). (*C*) Neutralization ability remained of NC solution and lyophilized NC–HA–sucrose powder stored at 4 °C for 30 d. (*D*) Photos of lyophilized powder, reconstituted solution, and inhalable spray. (*E*) Scheme illustrating the usage of hACE2 NC–containing powder. All data are presented as mean ± SEM (*n* = 3). The data are analyzed by Tukey’s multiple comparisons test. ns, *, **, and *** indicate no statistical difference, *P* < 0.05, *P* < 0.01, and *P* < 0.001, respectively.

For biological products such as antibodies and vaccines, it is necessary to adopt complex storage and preservation process to reserve their biological activities. For example, mRNA vaccines should be preserved at −20 °C to maintain their functions. Of note, lyophilized powder with cryoprotectant usually could be stored for years without losing its efficacy (32, 33). Thus, three commonly used cryoprotectants—sucrose, trehalose, and mannitol—were usually used as the candidates to prepare the dry formulation of NCs. Firstly, we studied the changes of NCs and the mixture of NC–HA with different cryoprotectants after freeze-drying and reconstitution in water. As shown in Fig. 3*A*, the size and zeta potential of NCs remained nearly unchanged in the presence of sucrose and trehalose, whereas NCs in control and mannitol groups showed obvious aggregation with increased sizes after being redissolved in water (Fig. 3*A*). We further optimized the freeze-drying conditions and found that NCs after fast freezing by liquid nitrogen exhibited higher neutralization ability than those with slow freezing by a freezing container (*SI Appendix*, Fig. S4). Compared with fresh NCs, NCs after freeze-drying retained their potency >90%, illustrating that sucrose is a promising cryoprotectant to prepare NCs in dry formulation (Fig. 3*B*). Excitingly, compared to NCs solution stored at 4 °C for one month, NC–sucrose lyophilized powder exhibited greatly remained neutralization ability (Fig. 3*C*). Thus, the NC–HA–sucrose formulation retained the ability of NCs to neutralize virus after freeze-drying, allowing for feasible usage and long-term storage (Fig. 3 *D* and *E*).

**Fig. 4.**
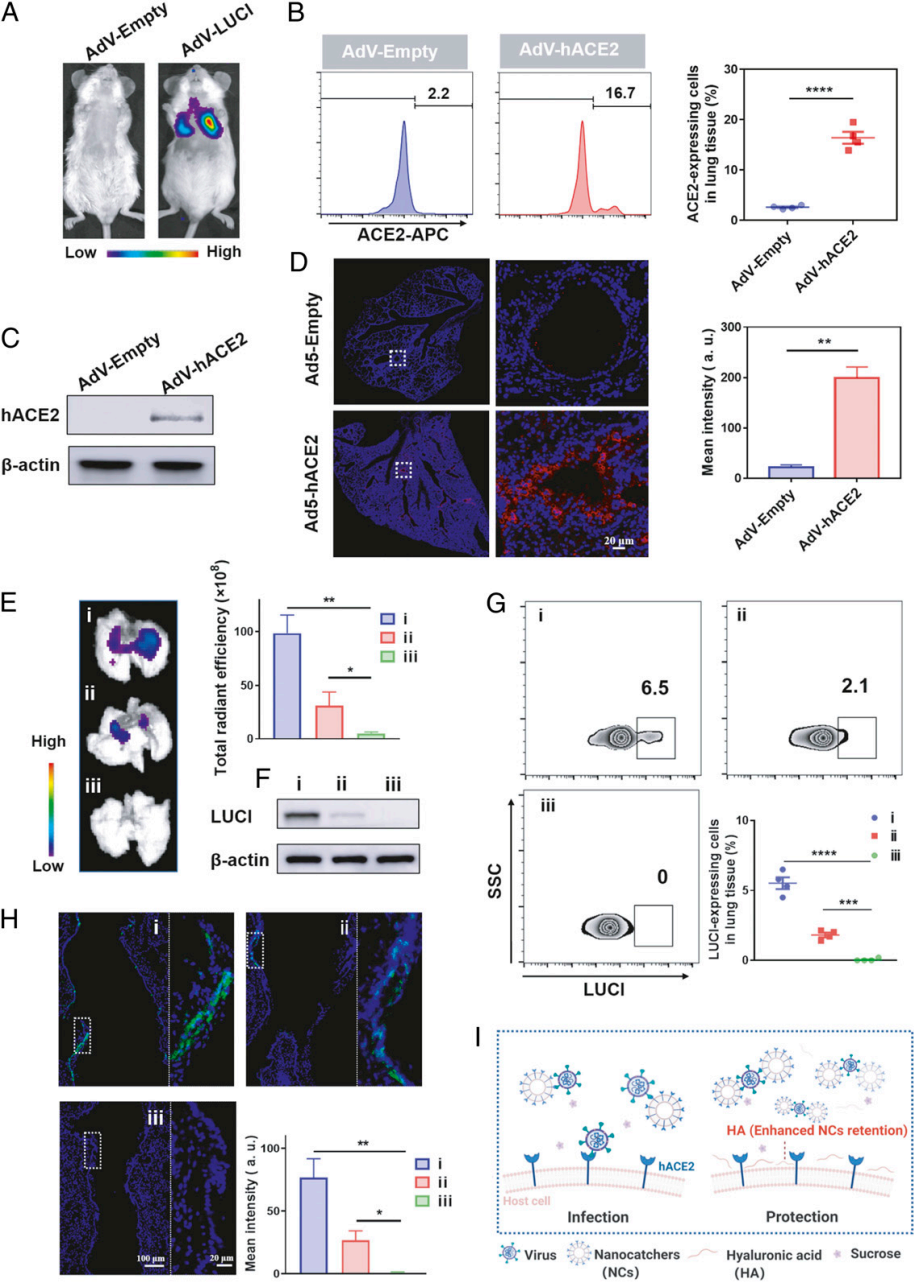
Inhibition of pseudotyped SARS-CoV-2 infection using the hACE2-expressing mouse model. (*A*) Bioluminescence image of mice inhaled with LUCI-encoding replication-defective AdV. (*B*) Representative flow cytometric image (*Left*) and related quantification results (*Right*), (*C*) Western blotting analysis, and (*D*) immunofluorescence images (*Left*) and related quantification analysis (*Right*) of AdV-Empty or AdV-hACE2–transduced lung tissues. (*E*) Bioluminescence imaging of lungs collected at 48 h post-PBS, NC–sucrose, and NC–HA–sucrose inhalation. (*Right*) is the quantified intensity of the bioluminescence intensity. (*F*) Western blotting, (*G*) flow cytometry, and (*H*) immunofluorescence analysis of lung tissues collected from mice after different treatments. (*i*), (*ii*), and (*iii*) represent PBS + pseudovirus, NC–sucrose + pseudovirus, and NC–HA–sucrose + pseudovirus group, respectively. (*Right*) is the enlarged image in (*Left*) white dotted frame (blue, nuclei; green, LUCI). (*I*) Scheme indicating the desirable protection effect of NCs against virus infection with the assistance of HA. All data are presented as mean ± SEM (*n* = 4). Data are analyzed by Tukey’s multiple comparisons test. *P < 0.05; **P < 0.01; ***P < 0.005; ****P < 0.001.

Before investigating the inhibition of SARS-CoV-2 infection in vivo, we first established the murine infection system, which is crucial to understand the infection process and evaluate the therapeutic results. Here, male nonobese diabetic/severe combined immunodeficient interleukin-2 receptor-γ chain null (NSG) mice were employed to construct the hACE2-expressing mouse model by inhalation delivery of replication-deficient adenovirus (AdV). NSG mice used in our experiment could reduce the clearance of virus by the immune system to achieve the desired AdV-hACE2 and pseudovirus infection effects (6, 34). LUCI-encoding AdV (AdV-LUCI, 1 × 10^10^ PFUs) were firstly employed to study the possibility of establishing such mouse model. Interestingly, obvious bioluminescence of LUCI in the lung was detected 5 d postinhalation, indicating that AdV could successfully induce the expression of specific proteins in the lung after inhalation (Fig. 4*A*). Subsequently, an NSG mouse was inoculated with the hACE2-encoding replication defective adenovirus (AdV-hACE2, 1 × 10^10^ PFUs) via the intratracheal route to construct the hACE2-expressing mouse model. The mRNA expression level of hACE2 in the lung was studied by the quantitative real-time PCR (q-PCR) assay at different days post–AdV-hACE2 administration, and a peak mRNA expression was observed at 5 d post administration (*SI Appendix*, Fig. S5). Then, the lung tissues of mice were excised for flow cytometry and Western blotting analysis at 5 d postadministration. Compared to the control group, the lungs of mice treated with AdV-hACE2 successfully expressed hACE2 at a high level (Fig. 4 *B* and *C*). Then, immunofluorescence staining was further used to demonstrate the expression of hACE2 in the lung. As shown in Fig. 4*D*, hACE2 was mainly distributed in the bronchus, further confirming the successful establishment of the hACE2-expressing mouse model by inhalation of AdV-hACE2.

Encouraged by the effective neutralization ability of NCs to inhibit the viral infection and the long-term lung retention of our inhalable NC formulation, we then evaluate the ability of NC–HA–sucrose to inhibit virus infection in vivo using the hACE2-expressing mouse model. Specifically, mice with hACE2 expression in the lung were divided into three groups, including phosphate-buffered saline (PBS) + pseudovirus, NC–sucrose + pseudovirus, or NC–HA–sucrose + pseudovirus. After being exposed to PBS, NC–sucrose, or NC–HA–sucrose, these mice were then subjected to pseudotyped SARS-CoV-2 at 4 and 8 h. As shown in the bioluminescence imaging, compared to the control group with strong bioluminescence of LUCI, which was from pseudovirus containing the LUCI reporter gene, moderate bioluminescence signals were observed in the NC–sucrose-treated group and nearly no signal was detected in NC–HA–sucrose-treated group, indicating that NC–HA with prolonged retention after inhalation could inhibit the virus infection in the lung (Fig. 4*E*). According to the results measured by flow cytometry, the percentage of LUCI-positive cells was 6.5, 2.0, and 0% for the control, NC–sucrose, and NC–HA–sucrose groups, respectively (Fig. 4*G*), which was consistent with the Western blotting analysis (Fig. 4*F*), further indicating the effective blockade ability of NC–HA–sucrose. Then, immunofluorescence staining visually displayed that nearly no fluorescence from the anti-LUCI antibody could be detected in the lung section collected from NC–HA–sucrose-treated mice, while obvious expression of LUCI infected by pseudovirus was observed in the bronchus of mice in the other two groups (Fig. 4*H*). Besides, no obvious difference was observed in pseudovirus- and HA + pseudovirus–treated lungs from the flow cytometry and immunofluorescence staining data, indicating that HA could not serve as a barrier to prevent virus invasion (*SI Appendix*, Figs. S6 and S7). Thus, with long-term lung retention after inhalation, hACE2-containing NCs together with mucoadhesive excipient HA exhibited potent pseudovirus inhibition in the hACE2-expressing mouse model (Fig. 4*I*).

**Fig. 5.**
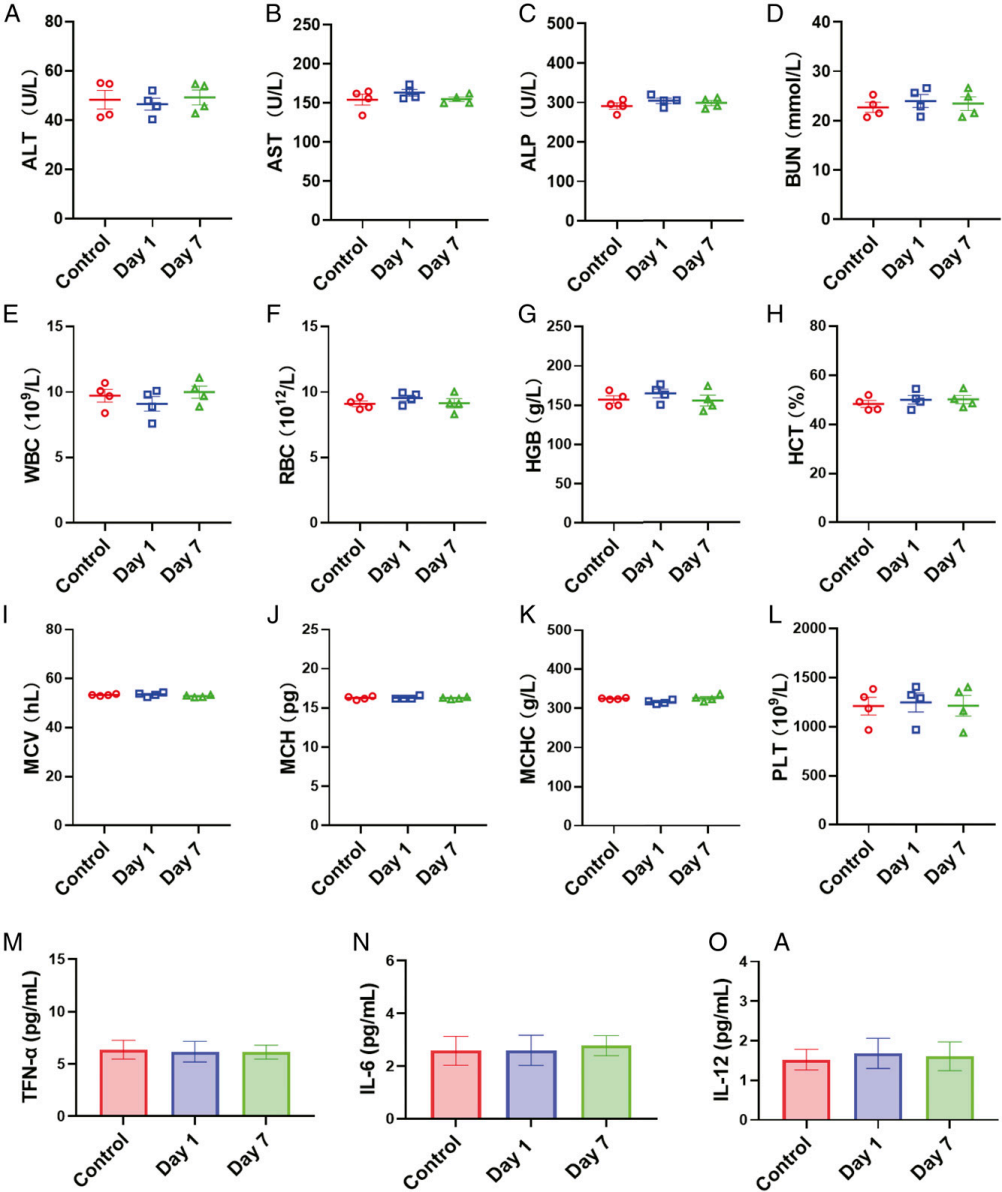
In vivo biosafety study of NC–HA–sucrose. (*A*–*D*) Blood biochemistry analysis. (*A*) ALT: alanine transaminase, (*B*) AST: aspartate aminotransferase, (*C*) ALP: alkaline phosphatase, and (*D*) BUN: blood urea nitrogen. (*E*–*L*) Complete blood tests. (*E*) WBC: white blood cell, (*F*) RBC: red blood cell, (*G*) HGB: hemoglobin, (*H*) HCT: hematocrit, (*I*) MCV: mean corpuscular volume, (*J*) MCH: mean corpuscular hemoglobin, (*K*) MCHC: mean corpuscular hemoglobin concentration, and (*L*) PLT: platelets. (*M*–*O*) Inflammatory cytokine detection. (*M*) TNF-α, (*N*) IL-6, and (*O*) IL-12 levels in the mouse sera were measured by ELISA. The data points represent mean ± SEM (*n* = 4).

After confirming the robust neutralization ability of NC–HA–sucrose against pseudotyped SARS-CoV-2, we also investigated their potential toxicity after inhalation in mice. Multiple blood parameters at day 1 and day 7 postinhalation were evaluated by serum biochemistry and complete blood test (Fig. 5 *A*–*H*). All these blood markers showed no significant difference from the PBS-treated group. Besides, the concentrations of serum inflammatory cytokine were all at baseline levels (Fig. 5 *I*–*L*), indicating no inflammation was induced by inhalation of NC–HA–sucrose. Moreover, hematoxylin and eosin (HE) staining revealed no pathological changes in major tissues at 7 d post-NC–HA–sucrose administration (*SI Appendix*, Fig. S8), confirming the nontoxicity of NC–HA–sucrose in vivo. All these results demonstrated that the excellent biocompatibility of the NC–HA–sucrose complex. Although more detailed data are still necessary to evaluate the systemic toxicity, our pilot toxicity study provides presumptive positive evidence supporting further development of these NCs for future clinical use.

## Discussion

In summary, we have developed a safe and effective inhalable hACE2-containing nanocatchers against COVID-19. The hACE2 NC-based platform offers many unique benefits over the existing treatments. Firstly, overexpressed hACE2 NCs could be potent agnostic to different viral mutations and potentially viral species by competing with host cells for virus infection. Such a strategy also could be extended to other potential epidemics. Secondly, the mucoadhesive excipient introduced in this system could significantly prolong the retention of NCs in the lung with desirable blockade effects. The successful inhibition of pseudovirus infection using the hACE2-containing cellular NVs was demonstrated in the hACE2-expressing mouse model. Thirdly, lyophilization with cryoprotectant endowed the NCs with long-term stability during storage without activity loss, significantly increasing the feasibility of their clinical use. Lastly, rapid and large-scale production of hACE2-containing NCs is feasible by using the engineered cell line. Therefore, our proposed strategy may have the potential to be implemented in the battle against COVID-19, especially in this urgent period with the frequent emergence of mutated viral strains.

## Materials and Methods

### Materials, Cell Lines, and Animals.

All chemicals were purchased from Sigma-Aldrich and used without any purification. HUVEC and 293T cells were obtained from the Cell Bank of the Chinese Academy of Sciences. Male Balb/c and NSG mice (8 to 10 wk old) were purchased from Nanjing Pengsheng Biological Technology Co. All animal experiments complied with the animal protection laws of China and were approved by the Soochow University Laboratory Animal Center (No. ECSU-2019000198).

### Preparation and Characterization of NCs.

Lentivector-encoding hACE2 with C-terminal monomeric mCherry tag (pLenti-C-mCherry-hACE2-puro) was purchased from Fubio Biological Technology Co., Ltd. To establish the stable cells, 293T cells were infected with lentivirus-encoding hACE2 (multiplicity of infection = 10) for 12 h and then were incubated with 2 μg/mL puromycin for selecting the cell line stably expressing hACE2 protein. 293T and hACE2-293T cells were cultured in Dulbecco’s modified Eagle medium (DMEM) (Gibco, Invitrogen) supplemented with 10% premium fetal bovine serum (Gibco) and 1% penicillin–streptomycin (Gibco) in a 5% CO_2_ incubator at 37 °C. Engineered 293T cells were seeded in the confocal dishes. The cell membrane dye DiO (5 μM) was conincubated with cells for 20 min in a cell incubator. Then, DiO was removed and washed with PBS three times for confocal laser scanning microscope (Zeiss) imaging in sequential scanning mode.

hACE2-293T cells were harvested with trypsin and suspended with homogenization medium (HM) containing 0.25 M sucrose, 1 mM EDTA, 10 mM Hepes (pH 7.4), and protease inhibitor mixture. The cells were disrupted by a POGSON92-II bath sonicator (Pogson) with a power of 200 W under an ice bath. Then, the suspension was centrifuged at 3,000 rpm for 10 min to remove cell nuclei and cytoplasm. The resulting packed cell membrane was washed once with cold HM solution. Afterward, cell membrane was collected by centrifugation at 14,800 rpm for 30 min. To prepare cell membrane NVs, the resulting suspension was extruded serially through 400- and then 200-nm polycarbonate porous membranes 10 times using an Avanti mini extruder (Avanti Polar Lipids). The size distribution and ζ-potential of nanocatchers were measured by a DLS instrument (Nano ZS ZEN3600, Malvern). The TEM image was observed by sample-deposited carbon-coated copper grids on JEM-1230 at an accelerating voltage of 80 kV. Membrane content was quantified by using a bicinchoninic acid protein assay (BCA) protein assay kit (Thermo Fisher) in reference to the bovine serum albumin (BSA) standard. Approximately 1 mg cell membrane (protein weight) could be collected from 2 × 10^8^ 293T cells.

### Western Blotting.

The protein contents were determined by a BCA kit. Equal amounts of protein solution were resolved in 2× Laemmli buffer (Sigma) and boiled at 95 °C for 5 min. After gel electrophoresis and protein transformation, proteins were analyzed by primary antibodies specific for β-actin antibody (Abcam, 1:5,000), angiotensin-converting enzyme 2 (ACE2; R&D, 1:20), and firefly LUCI (Abcam, 1:1,000) along with horseradish peroxidase–conjugated secondary antibody against goat IgG or rabbit IgG (Biolegend, 1:1,000). These blots were detected by enhanced chemiluminescence (Thermo Scientific).

### Flow Cytometry.

After being infected by pseudovirus, the lungs from mice were collected and made into single-cell suspensions, fixed with IC Fixation Buffer (Invitrogen), and permeated with 1× permeabilization buffer (Invitrogen). To confirm the ACE2 protein expression, cells were stained with goat anti-human ACE2 primary antibody (R&D, 1:20) at 4 °C for 1 h and then stained with DL649-labeled rabbit anti-goat secondary antibody (Abbkine, 1:500) for 30 min at 4 °C. AF647-conjugated anti-firefly LUCI antibody (Abcam, 1:100) was used for LUCI observation following the manufacturer's instructions. All the possessed cells were analyzed by flow cytometry (BD Accurit TM C6 Plus).

### Immunofluorescent Analysis of hACE2-293T.

After being infected by pseudovirus, cells were fixed in 4% paraformaldehyde, blocked, and permeabilized with 0.2% Triton X-100 in 2% BSA buffer. After that, cells were incubated with primary firefly LUCI antibody (Abcam, 1:500), followed by secondary antibody goat anti-rabbit IgG (FITC) (Abcam, 1:1,000). The nucleus was then stained with DAPI (Thermo Fisher Scientific) for 20 min.

### Immunofluorescent Analysis of Lung.

The lung tissue was fixed in 4% paraformaldehyde overnight, dehydrated in sucrose gradient solution, cryogenically embedded in optimal cutting temperature compound, and sectioned into 8-μm-thick slices. The sections were then blocked with 2% BSA and incubated with primary anti-ACE2 antibody (R&D, 1:200) or primary anti-firefly LUCI antibody (Abcam, 1:500) in 0.2% Triton X-100 in 2% BSA buffer at 4 °C overnight. Then, the sections were stained with DL649-labeled rabbit anti-goat secondary antibody or goat anti-rabbit IgG (FITC) (Abcam, 1:1,000) secondary antibody for 2 h at room temperature. Finally, the sections were stained with DAPI and observed in the confocal microscope. The imaging parameters were kept constant for different groups. Each section was quantified by ImageJ software.

### Neutralization Assay.

SARS-CoV-2-S-VSVΔG pseudotypes were purchased from Beijing Sanyao Science and Technology Development Co. The VSV pseudovirus system contains the S protein of SARS-CoV-2 and the LUCI reporter gene. A neutralization assay based on the pseudotyped virus was performed by measuring the infection of hACE2-293T cells. Briefly, 10^4^ cells/well in 100 μL were seeded in the 96-well plates and cultured for 16 h. Diluted NC-based inhibitors (50 μL) were incubated with an equal volume of SARS-CoV-2-S-VSVΔG pseudotypes (500 TCID_50_ [median tissue culture infective dose]) for 1 h at 37 °C, and then 50 μL mixture was transferred to hACE2-293T monolayers in 50 μL media, then further incubated at 37 °C for 48 h. Signal of LUCI was then read on a Luminometer (Synergy H1, Biotek) with LUCI substrate according to the manufacturer’s instructions (Promega). Neutralization efficiency (%) = [1 − (Signal of sample − Averaged signal of background)/(Averaged signal of virus-only control − Averaged signal of background)] × 100%. The IC_50_ were determined by four-parameter logistic regression (GraphPad Prism 8.3.0)

### Preparation and Characterization of Dry Formulation.

The mixtures of NCs (3 mg/mL), HA (1.5 mg/mL), and cryoprotectants (25 mg/mL) were prepared and frozen using either a fast or slow freezing process before lyophilization. The weight percentage of NCs in the lyophilized formulation was about 10.2%. For the fast-freezing process, the mixture was frozen rapidly using liquid nitrogen and then dried using a lyophilizer. For the slow-freezing process, the mixture was frozen using a Mr. Frosty freezing container at a cooling rate of −1 °C min^−1^ (Thermo Fisher Scientific) and then dried using a lyophilizer. Lyophilized samples were stored at 4 °C and reconstituted simply by water (molecular biology grade, Sigma).

### Lung Retention and Biodistribution.

Cy5.5-labeled NVs (NCs-Cy5.5) were prepared as previously depicted. Cy5.5-labeled NCs (100 μg) in 50 μL PBS buffer with or without HA (50 μg) were intratracheally delivered into male Balb/c mice (8 to 10 wk old) via inhalation by the microsprayer aerosolizer (BJ-PW-M, BioJane). Major organs were collected for fluorescence imaging at 6, 12, and 24 h postinhalation. Regions of interest were quantified as the radiance (photons s^−1^ ⋅ cm^−2^ ⋅ sr^−1^) using Living Image software. Then, the lung was collected and cut into tissue slices for confocal microscope imaging, and the intensity of fluorescence was calculated by ImageJ software.

### Animal Model Construction.

Male NSG mice (8 to 10 wk old) were anesthetized, and transduction with 1 × 10^10^ plaque-forming units (PFU) of a replication-defective adenovirus (AdV) encoding for LUCI in 50 μL DMEM was performed intratracheally three times with a time interval of 4 h. After 5 d, mice received an intraperitoneal injection of luciferin (Xenolight, PerkinElmer) at a dose of 0.2 mg/g and were observed using an IVIS Spectrum Imaging System (PerkinElmer) to confirm the expression of luciferin. Then, the hACE2-expressing mouse model was established in a similar way by intratracheal transduction with AdV-hACE2 three times with a time interval of 4 h. The expression of hACE2 was characterized by flow cytometry, Western blotting, and immunofluorescence staining.

### Inhibition of Pseudotyped SARS-CoV-2 Infection In Vivo.

hACE2-expressing NSG mice were randomly divided into three groups for intratracheal administration of PBS (50 μL), NC (3 mg/mL)–sucrose (25 mg/mL) (50 μL), or NC (3 mg/mL)–HA (1.5 mg/mL)–sucrose (25 mg/mL) (50 μL). Mice were challenged with pseudotyped SARS-CoV-2–containing LUCI reporter gene (1 × 10^6^ TCID_50_/mL) at 4 (50 μL) and 8 h (25 μL) postinhalation. Bioluminescence imaging, flow cytometry, Western blotting, and immunofluorescence staining were performed to evaluate the blockade effects of these groups.

### Biosafety Study.

The mice were inhaled with NC–HA–sucrose at the highest feasible dose of NCs (200 μg, based on membrane protein, in a suspension of 50 μL). At 1 and 7 d postinhalation, the mouse serum and whole blood samples were collected for analysis. The concentrations of inflammatory cytokine in the mouse sera were determined by enzyme-linked immunosorbent assay (ELISA) kits (eBioscience) according to the manufacturer’s guidelines.

### Statistical Analysis.

All results are presented as means ± SEM as indicated. Student’s *t* test and Tukey post hoc test were used for two-group and multiple comparisons, respectively. Statistical analysis was performed using GraphPad Prism software version 8.3.0.

## Supplementary Material

Supplementary File

## Data Availability

All data supporting the findings of this study are available within the article and *SI Appendix*.
