# Supplementary material for: Inhalable nanocatchers for SARS-CoV-2 inhibition
Source: Proc Natl Acad Sci U S A. 2021 Jul 2;118(29):e2102957118. doi: 10.1073/pnas.2102957118 (PMC8307760; doi:10.1073/pnas.2102957118)
Supplement: Supplementary File [file pnas.2102957118.sapp.pdf]

PNAS
www.pnas.org

Supplementary Information for

Inhalable Nanocatchers for SARS-CoV-2 Inhibition

Han Zhang[1], Wenjun Zhu[1], Qiutong Jin[1], Feng Pan[2], Jiafei Zhu[1], Yanbin Liu[1], Linfu Chen[1], Jingjing Shen[1], Yang Yang [2,3], Qian Chen[1]*, Zhuang Liu[1,4]**

1. Institute of Functional Nano & Soft Materials (FUNSOM), Jiangsu Key Laboratory for Carbon-Based Functional Materials & Devices, Soochow University, Suzhou 215123, China

2. Department of Thoracic Surgery, Shanghai Pulmonary Hospital, Tongji University School of Medicine, Shanghai 200433, China

3. School of Materials Science and Engineering, Tongji University, Shanghai, 201804, China

4. Macao Institute of Materials Science and Engineering, Macau University of Science and Technology, Taipa, 999078 Macau SAR, China

Corresponding e-mail: timyangsh@tongji.edu.cn; chenqian@suda.edu.cn; zliu@suda.edu.cn.

This PDF file includes:

Supplementary text

Figures S1 to S7

Supplementary text

Cytotoxicity Assay

Cytotoxicity of nanocatchers (NCs) to 293T and HUVEC cells were evaluated by methyl thiazolyl tetrazolium (MTT). Cells with a density of 1×10^4 cells/well were seed in 96-well plates and cultured at 37 °C for 16 h. After adding NCs of indicated concentrations, cells were incubated with NCs at 37 °C for 24 h. Before the measurement, MTT solution (5 mg/mL, 10 μL) and DMSO (100 μL) were pipetted into the medium sequentially. Cell viability was defined as the percentage of live cells per total control cells.

ACE2 RNA Analysis

q-PCR was performed using FastStart Universal SYBR Green Master (Rox) (Servicebio) by Stepone plus (ABI) in accordance with the instructions provided by the manufacturer. The following primers were used (5′→3′): M-GAPDH-S, CCTCGTCCCGTAGACAAAATG; M-GAPDH-A, TGAGGTCAATGAAGGGGTCGT; M-ACE2-S, ATCATCAAGCGTCAACTACAGGC; M-ACE2-A, TCGTTTTTCAGGA-CCACATACTCT. RNA expression was normalized to GAPDH expression in the relevant untreated controls.

Supplementary Figures

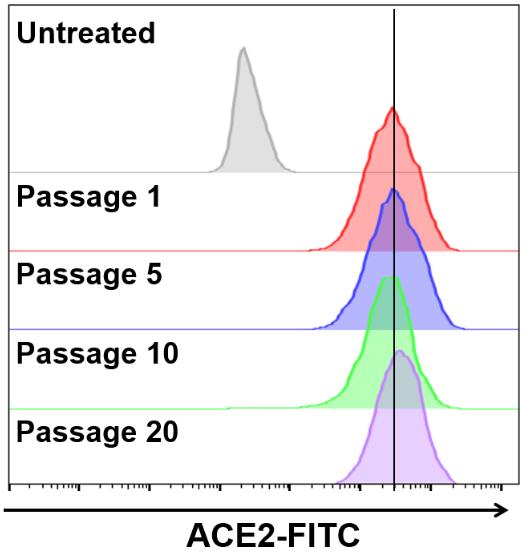

Figure S1. Flow cytometric analysis of hACE2 expression levels on hACE2-293T cells with different passage numbers.

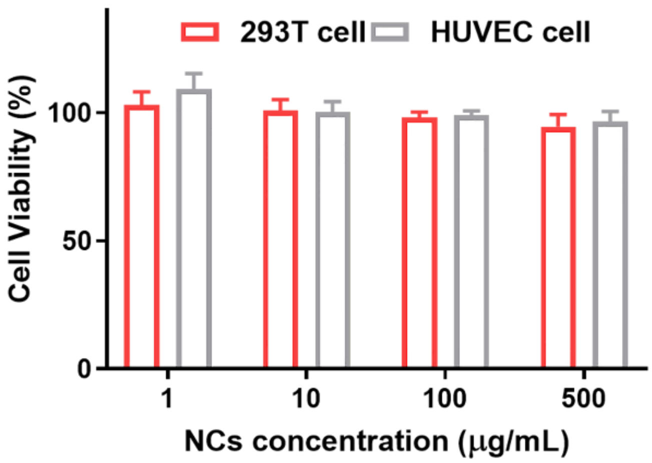

Figure S2. Relative viabilities of 293T and HUVEC cells after treatment with different concentrations of NCs. Data points represent as mean ± s.e.m. (n = 3).

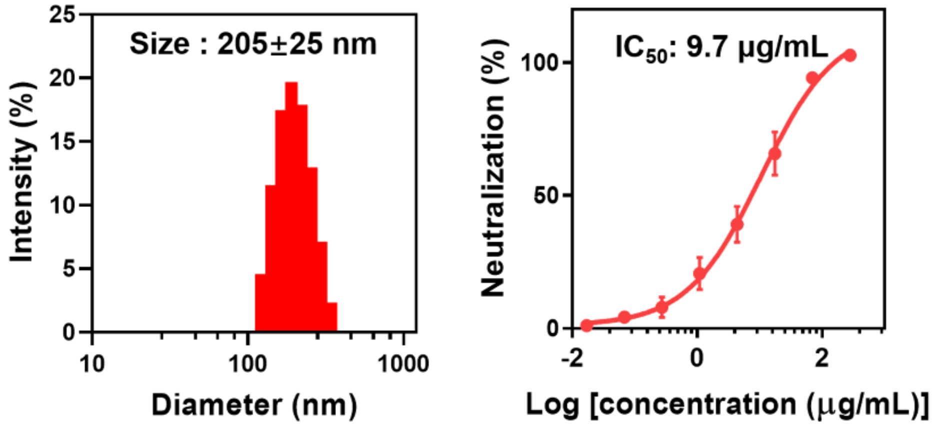

Figure S3. The size distribution (left) and pseudotyped SARS-CoV-2 neutralization curves (right) of NCs/HA. The zeta potential was measured to be about -16 mV.

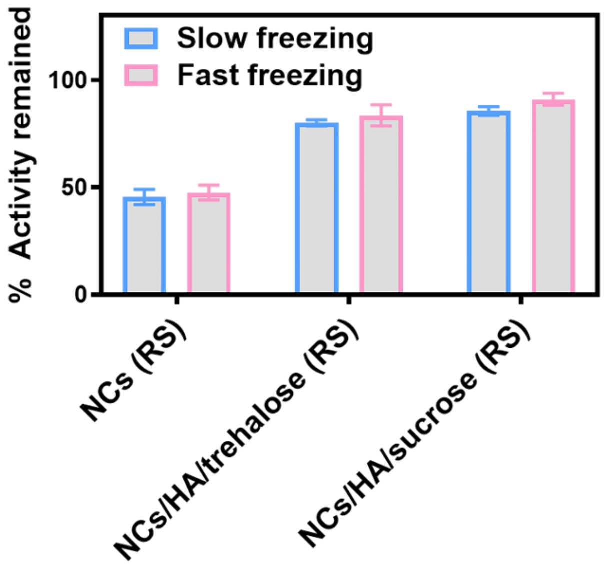

Figure S4. Relative pseudovirus neutralization efficiency of reconstituted NCs, NCs/sucrose and NCs/trehalose solutions compared to freshly prepared NCs sample (equivalent NCs concentration, 10 μg/ml) after slow or fast freezing. The pseudovirus neutralization efficiency of freshly prepared NCs solution is defined as 100%.

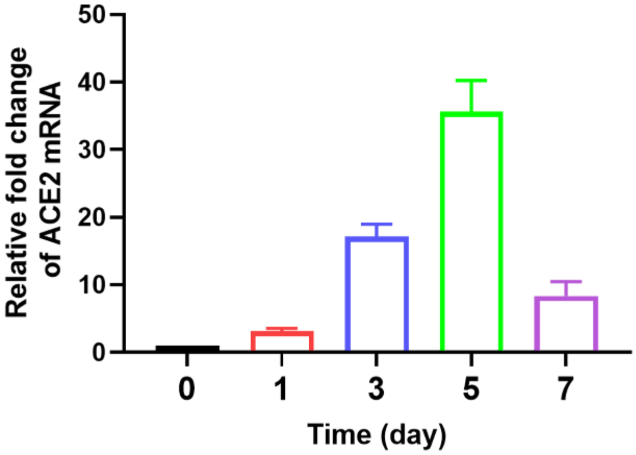

Figure S5. q-PCR analysis of ACE2 mRNA expression levels in lungs at the 1st, 3rd, 5th, and 7th day post AdV-hACE2 administration.

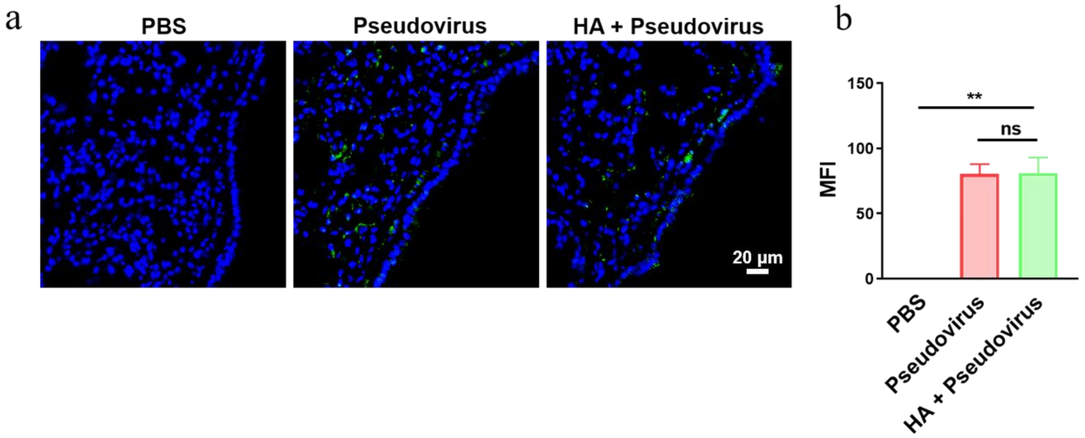

Figure S6. (a) Representative immunofluorescence imaging and (b) mean fluorescence intensity (MFI) of LUCI expressed in the lungs of mice at 24 h post-pseudovirus infection (blue, nuclei; green, LUCI). The Lung tissue in (a) is near the bronchus. Data points represent as mean ± s.e.m. (n = 5). Data are analyzed by Tukey's multiple comparisons test. Ns, and ** indicate no statistical difference and $P < 0.01$ respectively.

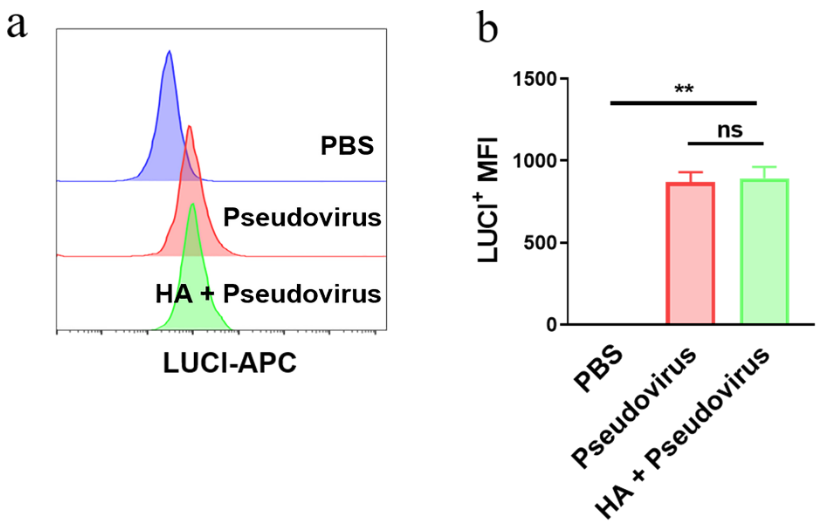

Figure S7. (a) Representative flow cytometry analysis and (b) MFI of LUCI^{+} lung cells of mice at 24 h post-pseudovirus infection. Data points represent as mean ± s.e.m. (n = 5). As compared with PBS treated group, ns and ** indicates no statistical difference and P < 0.01, respectively.
